# Neurocognitive performance and quality of life of older adults with HIV on antiretroviral treatment in Northern Thailand

**DOI:** 10.1002/jia2.25983

**Published:** 2022-09-29

**Authors:** Linda Aurpibul, Patumrat Sripan, Arunrat Tangmunkongvorakul, Wilawan Chaikan, Saowalak Sarachai, Kriengkrai Srithanaviboonchai

**Affiliations:** ^1^ Research Institute for Health Sciences Chiang Mai University Chiang Mai Thailand; ^2^ Faculty of Medicine Chiang Mai University Chiang Mai Thailand

**Keywords:** neurocognitive, quality of life, older adults with HIV, antiretroviral treatment, Thailand, cohort studies

## Abstract

**Introduction:**

With virologic suppression and longer life expectancy, older adults with HIV (OAHIV) are at risk for neurocognitive impairment (NCI). This study investigated neurocognitive performance, quality of life (QOL) and the association between OAHIV determinants.

**Methods:**

This cross‐sectional study was conducted in OAHIV aged ≥ 50 years on antiretroviral treatment at community hospitals in Northern Thailand between September and November 2020. The Montreal Cognitive Assessment Thai Version (MoCA‐T) and the Thai‐validated Medical Outcomes Study HIV (MOS‐HIV) were used. NCI was defined as MoCA‐T scores <25: 16–24 for amnestic mild cognitive impairment (aMCI) and <16 for dementia. For QOL, higher scores meant better QOL; a physical health summary T‐score ≥50 was defined as good QOL.

**Results:**

Overall, 269 OAHIV were enrolled; 59% were female and 99% had virologic suppression. The current median age was 61.8 years (interquartile range [IQR] 58.9–65.7). The median duration of antiretroviral treatment was 10.5 years (IQR 8.5–13.5). The current median CD4 count (234 tested) was 484 cells/mm^3^ (IQR 339–634), and 99% had plasma HIV RNA <40 copies/ml (229 tested). The median MoCA‐T score was 20.0 (IQR 16.3–23.0). There were 234 OAHIV (87.3%) with NCI: 182 (67.9%) with aMCI and 52 (19.4%) with dementia. A hundred and ninety (70.6%) had good QOL. Bivariate analysis revealed no correlation between MoCA‐T scores and QOL. Multivariable linear regression analysis revealed that MoCA‐T score was associated with older age (*r* = –0.144, *p* = 0.002), lower education (*r* = 0.629, *p* < 0.001), lower income (*r* = 0.797, *p* = 0.040) and shorter treatment duration (*r* = 0.189, *p* = 0.006).

**Conclusions:**

The vast majority of OAHIV with virologic suppression had NCI. Approximately two‐thirds had a mild impairment and one‐fifth had dementia. Neurocognitive performance and QOL were not correlated. Addressing mild NCI would enable more targeted monitoring. Early intervention and support could minimize functional impairment with increased age.

## 1 INTRODUCTION

Due to the widespread use of effective antiretroviral treatment (ART), the median age of people with HIV (PWHIV) has increased [1]. In 2016, an estimated 5.7 million PWHIV globally were over the age of 50 [2] accounting for 30% of adults with HIV in high‐income countries, and varying from 6% to 17% in other regions of the world [3]. In Thailand, the national scale‐up of HIV care began in 2000 [4]. Many patients on ART have survived into their fifties, and are now considered older adults with HIV (OAHIV). Apart from age‐related illnesses, studies have demonstrated an increased risk of chronic non‐AIDS‐related diseases, including neurocognitive complications in OAHIV, partly related to chronic inflammation [5].

Neurocognitive impairment (NCI) is one among several comorbidities associated with a decline in activities of daily living, cognitive symptoms and functional status in PWHIV [6]. NCI classifications include asymptomatic NCI, mild neurocognitive disorders and HIV‐associated dementia [7]. Prior to the widespread availability of ART, the prevalence of NCI in ART‐naïve adults with HIV in an Indian study between 2003 and 2004 was 60% [8]. NCI remained prevalent in the initial ART era, though profound HIV‐associated neurocognitive disorders were only infrequently seen [9]. A report from a Southeast Asian consortium study in 2006 documented moderate to severe HIV‐related NCI in 12% of PWHIV in both ART‐naïve and ART treatment groups [10]. The 2NN study in Thailand in 2008 documented NCI in 37.5% of PWHIV at a median age of 41.2 years who had good virologic control [11]. More recently, a 2015–2017 study in Bangkok reported an NCI prevalence of 59.4% among PWHIV with a median age of 54.3 years [12]. Factors associated with NCI included increased age, being female, low education, depression and low income [13, 14]. It is possible that pre‐existing neurological deficits were present prior to ART initiation, or that NCI developed later due to ongoing inflammatory processes associated with chronic HIV infection [15]. The consequences of NCI include poor medical adherence, disruption of daily functioning, decreased quality of life (QOL) and increased risk of mortality [16]. The impact of NCI on health‐related QOL of PWHIV in all domains was documented [17]. Meanwhile, a Kenyan study reported worse QOL in PWHIV without significantly different cognitive deficits [18]. There have been several studies of QOL among PWHIV in Thailand. However, an association between QOL and NCI has not been well established [19, 20].

In this study, we hypothesized that even with sustained virologic suppression through ART, OAHIV could remain at increased risk for impaired QOL and early NCI. The study objective was to determine the frequency of NCI among Thai OAHIV residing in communities. We assessed the correlation between neurocognitive performance and QOL and looked for factors associated with NCI in this population.

## 2 METHODS

### 2.1 Study design and population

A cross‐sectional study was conducted at 12 community hospitals in Chiang Mai, Thailand between September and November 2020. The study population was OAHIV in a prospective cohort study, which was started in August 2015. The primary aim of the cohort is to follow the QOL and health outcomes of OAHIV receiving the national standard of HIV care. The national AIDS programme covered antiretroviral medication, CD4 counts and laboratory safety parameter measurements every 6 months, and annual HIV‐RNA testing. Treatment for comorbidities and other diseases is covered under the universal healthcare coverage scheme for all Thai citizens. In every community hospital, an HIV clinic had been set up that was separated from the outpatient services for general patients, which afforded greater privacy and confidentiality. For this cohort, 12 hospitals were selected from a total of 23 community hospitals in the province, according to the advice of the Chiang Mai Provincial Public Health office. Specifically, the selected hospitals had been serving larger populations with HIV since the national HIV programme was launched in 2002. In 2015, potential participants were approached and invited to join the cohort study during routine clinical visits. The inclusion criteria were (1) living with HIV, (2) being aged ≥50 years and (3) receiving ART at study enrolment. Each hospital approached its oldest patients first. After enrolment, all participants were continuously followed in HIV clinics for regular care at 2‐ or 3‐month intervals under the Thai national AIDS programme. All participants who attended their annual follow‐up visits in 2020 were invited to join this study.

### 2.2 Sample size estimation

The prevalence of NCI among PWHIV and non‐HIV older adults in other studies were between 59.4% and 71.4% [12, 21]. In our study, the estimated prevalence was 80%. The evaluable number of participants was 246, which would have 80% power at the confidence interval of 95% to assess NCI.

### 2.3 Ethics statement

The study was approved by the Ethics Committee at the Research Institute for Health Sciences, Chiang Mai University (Certificate approval number 35/2020). Written informed consent was obtained from each participant. The study staff assessed their literacy prior to the consent process (e.g. through reading out loud). Literate participants gave written consent, while low literacy and illiterate participants provided consent with a thumbprint in presence of an impartial witness.

### 2.4 Data collection and tools

A range of demographic characteristics (sex, age, education, monthly income, number of family members and year of HIV diagnosis) were obtained as part of the main study. Clinical data (date of ART initiation, current CD4, HIV RNA and comorbidities) were extracted from hospital records by HIV clinic staff. After consent, the assessment took place in a private space within the hospital. Cognitive performance was assessed by certified investigators (physicians and nurses) using the Thai version of the Montreal Cognitive Assessment (MoCA‐T). MoCA‐T is a screening measure that covers eight cognitive domains, including attention and concentration, visuospatial/executive function, memory, language, visuoconstructional skills, conceptual thinking, calculations and orientation. MoCA‐T has been validated with acceptable reliability for use in screening for amnestic mild cognitive impairment (aMCI) in Thai patients. The internal consistency was demonstrated to have a Cronbach's alpha coefficient of 0.914 [13]. MoCA‐T has also been used in studies among PWHIV after ART initiation [12, 14]. When an assessment was completed, all scores were summed out of a total possible score of 30. For individuals with ≤6 years of education, one point was added to their summed score. In this study, NCI was defined as a MoCA‐T score <25. Scores 16–24 were defined as aMCI, and scores <16 as dementia. With these cut‐offs, the areas under the receiver‐operating characteristics (ROC) curve were 0.813 for aMCI and 0.938 for Alzheimer's disease compared to healthy controls in a psychometric properties study [22].

Health‐related QOL was assessed using the Thai‐validated version of the Medical Outcomes Study HIV (MOS‐HIV) [23]. It was developed from the MOS‐Short form 20 with additional constructs relevant to PWHIV, and has been used in HIV‐related clinical trials as an outcome measure [24]. It consists of 35 close‐ended items measuring 11 dimensions of health‐related QOL, namely general health perception, physical functioning, role functioning, social functioning, cognitive functioning, pain, mental health, energy fatigue, health distress, QOL and health transition. For each question, participants would provide their responses in 2‐ to 6‐point Likert scales. Some questions were in the positive direction, and some were reversed. The dimensions are scored ranging from 0 to 100. Higher scores indicate better perceived QOL. The dimensions are then aggregated into the physical health summary (PHS) and mental health summary (MHS) status scores, in which they are linearly transformed to a 0–100 scale using factor‐analysis‐based or regression‐based weights. The standardized scores (T‐scores) had a mean of 50 and a standard deviation of 10. The norm‐based standardization dimension scores, the PHS and MHS T‐scores, were reported. Higher dimension scores meant better QOL. Based on the Norm‐Based Scoring, a PHS T‐score of ≥50 was defined as a good QOL [25].

### 2.5 Statistical analysis

Statistical analysis was performed using SPSS version 22.0 (IBM Corporation, New York). Demographic characteristics were presented by numbers (percentages) for categorical data, means (standard deviation, SD) for continuous data with normal distributions and medians (interquartile range [IQR]) for continuous data with non‐normal distributions. A comparison of demographic characteristics between OAHIV with impaired and good QOL was made using the chi‐square test or Fisher's exact test, as appropriate for categorical variables, and Mann–Whitney tests for continuous variables. The outcome variables of interest in this study were total MoCA score and domain scores, the proportion of participants with NCI (aMCI and dementia), PHS and MHS T‐scores, and itemized scores in 11 dimensions of QOL. Bivariate correlation between each score was analysed. Linear regression was used to identify factors potentially associated with MoCA scores: age, sex, education, time since HIV diagnosis, time from HIV diagnosis to ART initiation, duration on ART, monthly income, CD4 at study entry, current CD4, marital status and presence of comorbidities. In the multivariable regression analysis, factors with statistical significance in univariable analysis or that had a potential role as confounders were included. Statistical significance was defined as *p* < 0.05.

## 3 RESULTS

### 3.1 Characteristics of study participants

Two hundred and sixty‐nine OAHIV were approached. All provided consent and were enrolled in this study. One hundred and fifty‐nine (59.33%) were female. QOL assessments were completed in 268, and one PWHIV was excluded due to insufficient literacy. Their median age was 61.8 years (IQR 58.9–65.7), and the median monthly income was 4000 Baht (IQR 2000–10,000; ∼125 US Dollars). Two hundred and thirteen (79.5%) received ≤4 years of formal education. The median duration from HIV diagnosis to ART initiation was 15.4 years (IQR 10.6–19.3); 233 (86.6%) started ART (non‐nucleoside reverse transcriptase‐based regimens) between 1998 and 2013 while they were immunosuppressed, and 36 (13.4%) started ART after 2013 when it became available for all at any CD4 level. The median ART duration at the time of this study was 10.5 years (IQR 8.5–13.5). The current median CD4 cell count was 484 cells/mm^3^ (IQR 339–634) of 234 tested, and 99% were virologically suppressed (HIV‐RNA <40 copies/ml) of 229 tested. Comorbidities were reported in 119 (44.4%) of the participants and included dyslipidaemia in 101 (37.7%), diabetes in 35 (13.1%) and renal diseases in 5 (1.9%). There was no difference in demographic characteristics between OAHIV with impaired and good QOL (Table 1).

**Table 1:** Demographic characteristics of all older adults with HIV in this study

Characteristics	Total	OAHIV with impaired QOL	OAHIV with good QOL	*p*‐value	Test statistics
Number of OAHIV	269	79 (29.4%)	190 (70.6%)		
Sex					
Female	159 (59.3%)	43 (27.0%)	116 (73.0%)	0.314	–1.004
Male	110 (40.9%)	36 (32.7%)	74 (67.3%)		
Age (years)	61.8 (58.9–65.7)	62.0 (59.0–66.0)	61.0 (59.0–65.3)	0.934	–0.083
Age > 60 years	160 (59.5%)	47 (29.4%)	113 (70.6%)	0.998	–0.003
Formal education					
0–4 years	213 (79.5%)	57 (72.7%)	156 (82.1%)	0.055	–2.260
5–12 years	46 (17.2%)	16 (20.3%)	30 (15.8%)		
> 12 years	10 (3.7%)	6 (7.6%)	4 (2.1%)		
Monthly income (Thai baht)	4000 (2000–10,000)	5000 (3000–10,000)	3800 (2000–9000)	0.056	–1.914
Marital status					
Married or in a relationship	106 (39.4%)	28 (35.4%)	78 (41.1%)	0.391	–0.856
Single/separated/divorced	163 (60.6%)	51 (64.6%)	112 (58.9%)		
Duration since HIV diagnosis (years)	26.2 (23.3–28.8)	26.6 (24.0–29.1)	25.9 (23.2–28.7)	0.522	–0.640
Duration on ART (years)	10.5 (8.5–13.5)	9.9 (8.1–13.1)	10.8 (8.5–13.6)	0.114	–1.581
Current CD4 cell count, cells/mm^3^ (*n* = 234)	484 (339–634)	468 (305–590)	501 (357–666)	0.227	–1.209
Current virologic suppression	227/229 (99.1%)	70/70 (100%)	157/159 (98.7%)	1.000	–0.940
Comorbidities	119 (44.4%)	32 (40.5%)	87 (45.8%)	0.427	0.793
Renal disease	5 (1.9%)	1 (1.3%)	4 (2.1%)	1.000	0.463
Diabetes	35 (13.1%)	11 (13.9%)	24 (12.6%)	0.774	–0.286
Dyslipidaemia	101 (37.7%)	27 (34.2%)	74 (38.9%)	0.462	0.734

Note: Data in median (interquartile range, IQR), mean (standard deviation, SD) or number (%) as appropriate. Impaired QOL was defined as a physical health summary (PHS) T‐score < 50, and good QOL was defined as PHS T‐score ≥ 50. *p*‐value by chi‐square, Fisher's exact or Mann–Whitney test.

Abbreviations: ART, antiretroviral treatment; OAHIV, older adults with HIV; QOL, quality of life.

### 3.2 Quality of life

The median PHS T‐score was 54.97 (IQR 48.07–58.46). There were 190 OAHIV (70.6%) with good QOL (PHS T‐score ≥50) and 79 (29.4%) with impaired QOL (PHS T‐score <50). The highest physical health dimension scores were role and social functioning, whereas the lowest dimension score was general health. The median mental health dimension (MHS) T‐score was 58.40 (54.30–61.74), with the highest score for health distress and the lowest dimension score was health transition. The PHS and MHS T‐scores were significantly correlated (Pearson's coefficient 0.522, *p* < 0.001). Those with good QOL also had a significantly higher median MHS T‐score (59.54 vs. 54.03 in those with impaired QOL, respectively; *p* < 0.001). When comparing groups, OAHIV with impaired QOL had significantly lower median scores than those with good QOL in all dimensions, except for the health transition domain. Scores were especially divergent in pain scores and in energy & fatigue (vitality) scores (Table 2).

**Table 2:** Neurocognitive performance and quality of life of OAHIV in this study

Characteristics	Maximum scores	Total	OAHIV with impaired QOL	OAHIV with good QOL	*p*‐value	Test statistics
*Quality of life (MOS‐HIV), n = 269*						
*Physical health summary T‐score*		**54.97 (48.07–58.46)**	**41.01 (33.75–46.84)**	**56.93 (54.59–59.43)**	**<0.001**	**–12.914**
General health	100	65.00 (55.00–82.00)	50.00 (35.00–65.00)	70.00 (60.00–80.00)		
Physical functioning	100	91.67 (75.00–100)	66.67 (41.67–75.00)	91.67 (83.33–100)		
Role functioning	100	100 (100–100)	50.00 (0–50.00)	100 (100–100)		
Social functioning	100	100 (100–100)	60.00 (40.00–100)	90.00 (80.00–100)		
Pain	100	77.78 (55.56–100)	44.44 (44.44–66.67)	88.89 (66.67–100)		
*Mental health summary T‐score*		**58.40 (54.30**–**61.74)**	**54.03 (49.02**–**58.97)**	**59.54 (56.49**–**62.75)**	**<0.001**	–**6.790**
Cognitive functioning	100	90.00 (80.00–95.00)	85.00 (75.00–90.00)	90.00 (85.00–95.00)		
Mental health	100	92.00 (80.00–100)	84.00 (76.00–96.00)	96.00 (80.00–100)		
Energy & fatigue (vitality)	100	85.00 (70.00–95.00)	65.00 (50.00–80.00)	90.00 (80.00–100)		
Health distress	100	100 (100–100)	90.00 (75.00–100)	100 (100–100)		
Quality of life	100	75 (75–75)	75.00 (50.00–75.00)	75.00 (50.00–100)		
Health transition	100	50.00 (50.00–75.00)	50.00 (50.00–75.00)	50.00 (50.00–75.00)		
*Neurocognitive performance (MoCA), n = 268*						
Median scores (IQR)	30	20 (16.3–23.0)	20.0 (16.0–24.0)	20.0 (17.0–22.0)	0.385	–0.868
Mean scores ±SD		19.3 ± 4.7	19.7 (5.0)	19.2 (4.6)	0.418	0.812
Domain scores						
Visuospatial/executive	5	2.9 (1.4)	2.9 (1.4)	2.9 (1.4)	0.810	0.241
Naming	3	2.5 (0.8)	2.5 (0.7)	2.4 (0.8)	0.390	0.862
Attention	6	4.3 (1.4)	4.3 (1.4)	4.3 (1.4)	0.853	0.186
Language	3	0.8 (0.9)	0.9 (0.9)	0.7 (0.8)	0.100	1.649
Abstraction	2	0.6 (0.8)	0.7 (0.8)	0.6 (0.8)	0.447	0.762
Delayed recall	5	1.8 (1.6)	1.9 (1.6)	1.8 (1.6)	0.489	0.693
Orientation	6	5.7 (0.7)	5.8 (0.6)	5.7 (0.8)	0.435	0.782
Neurocognitive impairment, *n* (%)		234 (87.3%)				
aMCI, *n* (%)		182 (67.9%)	48 (60.8%)	134 (70.9%)	0.052	1.499
Dementia, *n* (%)		52 (19.4%)	15 (19.0%)	37 (19.6%)		

Note: MoCA, the Thai version of the Montreal Cognitive Assessment; MOS‐HIV, the Thai‐validated version of the Medical Outcomes Study HIV; NCI, neurocognitive impairment (scores < 25); aMCI, amnestic mild cognitive impairment (scores 16–24); dementia (scores <16). Bold values highlights that they are summary T‐scores, which are significantly different between the two groups.

Abbreviations: IQR, interquartile range; OAHIV, older adults living with HIV; SD, standard deviation.

### 3.3 Neurocognitive performance

During the assessment, all participants were asked to complete each test consecutively without skipping. The median MoCA score was 20.0 (IQR 16.3–23.0). The median MoCA scores were not different between OAHIV with good and impaired QOL (*z* = –0.868, *p* = 0.385). The three domains with the lowest scores included language, abstraction and delayed recall. There were 234 OAHIV (87.3%) with NCI (score <25). A hundred and eighty‐two (67.9%) had aMCI (scores 16–24), whereas 52 (19.4%) had dementia (scores <16) (Table 2).

### 3.4 Correlation

The bivariate correlation revealed no relationship between MoCA and PHS T‐score (*r* = 0.030, *p* = 0.627), MHS T‐score (*r* = 0.033, *p* = 0.587) or cognitive functioning dimension score (*r* = 0.006, *p* = 0.925) (Table 3). Comparison between OAHIV with dementia and those with better neurocognitive performance (MoCA scores <16 vs. ≥16) also revealed no significant differences in PHS T‐score (*z* = –1.736, *p* = 0.083) and MHS T‐scores (*z* = –0.506, *p* = 0.614), or any QOL dimension scores.

**Table 3:** Bivariate correlation between MoCA and quality of life scores in older adults with HIV (*n* = 268)

Quality of life dimensions	Correlation coefficients^a^	*p*‐value	Median^b^	Test statistic (U)	*p*‐value
*Physical health summary T‐score*	0.030	0.627	54.97	7981.0	0.796
General health	0.052	0.395	65.00	8072.5	0.914
Physical functioning	0.098	0.108	91.67	7363.0	0.188
Role functioning	0.007	0.909	100.00	7749.5	0.446
Social functioning	–0.041	0.503	100.00	7629.5	0.257
Pain	0.047	0.444	77.78	7958.0	0.762
*Mental health summary T‐score*	0.033	0.587	58.40	7816.0	0.595
Cognitive functioning	0.006	0.925	90.00	7756.5	0.521
Mental health	–0.014	0.824	92.00	7754.5	0.519
Energy & fatigue (vitality)	0.038	0.539	85.00	8095.0	0.943
Health distress	0.041	0.506	100.00	7986.0	0.778
Quality of life	0.032	0.599	75.00	7824.0	0.580
Health transition	–0.023	0.708	50.00	8008.5	0.806

Note: Data in median (IQR) or number (%).

^a^ Pearson correlation estimates.

^b^ Comparison between those with dementia (MoCA score < 16) and those with normal or mild impairment (score ≥16).

### 3.5 Factors associated with NCI

The univariate linear regression revealed that age, education, ART duration and income were associated with MoCA scores, whereas sex, duration of HIV or treatment, CD4 at study entry, current CD4 and comorbidities were not associated. In the multivariable analysis, sex and comorbidities were specifically included as potential determinants of NCI. We found positive association with MoCA scores for years of education (β = 0.629, *p* < 0.001), ART duration (β = 0.189, *p* = 0.006) and monthly income (β = 0.797, *p* = 0.040), while age was negatively associated (β = –0.144, *p* = 0.002) (Table 4).

**Table 4:** Univariable and multivariable linear regression analysis of factors associated with MoCA scores of OAHIV

Covariate	Univariable			Multivariable*	
	*R*	β coefficient (95% CI)	*p*‐value	β coefficient (95% CI)	*p*‐value
Sex	0.079	0.753 (–0.399 to 1.905)	0.199	0.307 (–0.698 to 1.312)	0.547
Age (years)	0.214	–0.187 (–0.291 to –0.084)	<0.001	–0.144 (–0.234 to –0.054)	0.002
Education (years)	0.470	0.678 (0.524–0.831)	<0.001	0.629 (0.475–0.783)	<0.001
Duration from HIV diagnosis	0.004	0.004 (–0.124 to 0.132)	0.951		
Time from HIV diagnosis to ART initiation (years)	0.089	–0.071 (–0.167 to 0.025)	0.146		
Duration on ART (years)	0.148	0.190 (0.036–0.343)	0.015	0.189 (0.056–0.322)	0.006
Current monthly income (Thai baht)	0.236	1.693 (0.852–2.534)	<0.001	0.797 (0.035–1.559)	0.040
CD4 at study entry (year 1)	0.002	0.003 (–0.126 to 0.131)	0.969		
Current CD4 (year 5)	0.036	0.036 (–0.002 to 0.003)	0.586		
Marital status	0.072	0.696 (–0.465 to 1.857)	0.239		
Comorbidities	0.074	–0.700 (–0.167 to 0.025)	0.089	–0.658 (–1.634 to 0.0317)	0.185

Abbreviations: ART, antiretroviral treatment; CI, confidence interval; MoCA, the Montreal cognitive assessment; OAHIV, older adults with HIV.

* Factors with statistical significance or potential role as confounders were included in the multivariable analysis.

## 4 DISCUSSION

More than two‐thirds of OAHIV in this study had good QOL, while 87.3% of them had NCI, including one‐fifth who had dementia. There was no correlation between their neurocognitive performance and QOL. Low MoCA scores were associated with older age, fewer years of formal education, shorter duration on ART and low income.

Recently, the MoCA‐T has been increasingly used to identify Thai PWHIV with NCI and is a user‐friendly tool for NCI screening in HIV clinics. The median MoCA score of our study participants was 20.0 (IQR 16.3–23.0), which was lower than the median MoCA score of 24 (IQR 21–26) reported in a previous study in Bangkok [12]. The frequency of NCI in their cohort of 340 OAHIV (median age of 54.3 years) with good immune status and virologic suppression was 59.4%, which was lower than in our study. Nearly one‐third of their participants had a bachelor‐level education or higher, were living with an HIV diagnosis for a median of 18.3 years (IQR 14.9–20.9) and had been taking ART for a median of 16.1 years. With similar immunologic and virologic outcomes, our participants were older, less educated and experienced a median of 15.4 years delay between HIV diagnosis and ART initiation.

A large majority (86.6%) of OAHIV in our study started ART before the test and treat era in 2013. During that period, ART was not prescribed for PWHIV under the National AIDS programme until they developed some degree of immunosuppression. Meanwhile, the Bangkok study enrolled participants from a long‐term study cohort in which ART was initiated approximately 2 years after the HIV diagnosis as a part of the clinical trial in 2002. The delayed ART initiation in our participants might explain the higher rate of NCI. Another neurocognitive study was conducted among Thai PWHIV with virologic suppression, at the mean age of 45 years and a median ART duration of 12.1 years, where the frequency of NCI was 43% [14]. Their mean MoCA score of 22.24 ± 3.83 was significantly lower than the HIV‐negative comparison group. Within the HIV group, worse performance was observed in those who were older and had lower incomes. No associations with HIV‐related characteristics were reported, which is in line with our study findings.

There was a study conducted in 2020 among older villagers in the rural area of Northern Thailand where the prevalence of aMCI was 71.4% [21]. The mean age of their participants was 68.3 years and 95.1% had formal education of ≤4 years, which was more similar to our participants. Apart from having HIV, the higher prevalence of NCI in our study could be affected by the diversity in geographic, ethnocultural and education factors. Despite having Thai citizenship, some participants were from ethnic minorities (Shan people, Yunnan and hill tribes) who may have had less access to formal education in childhood. In the present study, we found low domain scores in language, abstraction and delayed recall, while in their study, the most common impairments were executive function, attention and delayed recall.

The impairment in delayed recall indicates impaired memory and is an early sign of AD, while impaired visuospatial/executive function indicates neurodegenerative change [26]. However, our participants did not perceive their memory problems (the median cognitive function domain score in QOL was 90/100). Moreover, they had a high role and social functioning (median scores 100/100). Low perceived difficulty in their daily activities might have been due to low instrumental activities involved. Our participants lived in communities with relatively low technology environments, where high‐level cognitive skills were not required. This may have been why their NCI did not affect their perceived QOL. The investigators who performed NCI assessment in our study reported language and abstraction as the most problematic domains.

Different MoCA score cut‐offs were mentioned across studies. In a US study, the frequency of NCI in OAHIV was 67% when a cut‐off of ≤26 was used. Their mean score was 25.2±3.0, the mean age of their participants was 58.2 years and the mean duration of HIV infection was 18 years; 98% were on ART and 92% were virologically suppressed [6]. A Malaysian study reported that 64.3% of PWHIV, mean age 44.7 years, had cognitive impairment using a MoCA cut‐off score of ≤26 [27]. However, when the adjusted T‐scores were applied instead of the standard scores, it resulted in decreased frequency of cognitive impairment in their study to 23.4%. Another study in Thailand was conducted in a tertiary care hospital among 74 PWHIV with a mean age of 45.6 years, about half were diagnosed with NCI using the Frascati criteria [28]. Their ROC analysis revealed that using a lower MoCA cut‐off of ≤23 instead of ≤24 would yield higher specificity of the MoCA in screening for NCI. As using different MoCA cut‐off results in varying frequencies of impairment, it is difficult to make direct comparisons between studies, and the results must be interpreted with caution.

Determination of QOL in PWHIV is critical as it allows healthcare providers to better understand their patients’ perceptions of their health and life satisfaction [29]. HIV programme assessment based on clinical outcomes alone may not be sufficient to ensure the wellbeing of OAHIV experiencing age‐related changes after years on ART. We found that two‐thirds of OAHIV in this study had good QOL. Those with good physical health (PHS) also had higher mental health (MHS) T‐scores. This was in line with a 2017 study in Northern Thailand which reported that 96.3% of PWHIV aged 20–60 years reported overall QOL at moderate levels, as assessed by the World Health Organization QOL brief assessment tool [30]. They mentioned that CD4 count <200 cells/mm^3^ was associated with low QOL. We did not see this association, as our participants had higher median CD4 counts at the time of the study. Another study in 2012 on PWHIV at a tertiary care hospital in Thailand also used the MOS‐HIV. Their population's median age was 42 years, the mean duration following HIV diagnosis was 6.8 years and 54% had been on ART for between 1 and 5 years [20]. They reported that age <50 years was a positive predictive factor for PHS while being on ART and having good compliance were positive predictive factors for MHS.

An earlier analysis of our cohort using data from 2015 to 2017 demonstrated that QOL in OAHIV could improve or be maintained over time [19]. Our participants were virologically suppressed, which indicated good ART compliance, and we did not see an age difference between those with good and impaired QOL. A Pakistani study reported that age >50 years, CD4 count >500 cells/mm^3^ and a primary/secondary education were associated with better QOL when compared to younger participants with a lower CD4 count, or those with tertiary education [31]. We found no significant difference in education or neurocognitive performance between OAHIV with impaired and good QOL. However, the observed high scores in the role and social functioning dimensions suggested that there might be other determinants of QOL that were stronger contributors (i.e. socio‐demographic, social support, geographic or environmental factors). Sex, marital status and physical exercise engagement were predictors of QOL in a study among Thai OAHIV [19]. A Kenyan study documented poorer QOL and mental health in PWHIV with low literacy in relation to the HIV‐negative group, despite exhibiting no significant cognitive deficits [18]. Living with the chronic disease might partly explain the low QOL, as we observed the lowest median scores in the pain, role functioning and energy & fatigue (vitality) domains in OAHIV with impaired QOL. Screening for frailty and addressing support needs could be useful interventions in this context.

The low median health transition scores in all study participants regardless of their QOL might have been impacted by the Covid‐19 pandemic. At the time of the assessment, the worsening Covid‐19 situation in Northern Thailand resulted in many new public health restrictions (e.g. social distancing, face masks and limited travel). Since the health transition question asked about physical health and emotional condition compared to the previous 4 weeks, these restrictions could have been a factor in their responses.

The study had several strengths. To our knowledge, this is the first neurocognitive study of community‐dwelling OAHIV in rural Thailand. Our study participants represented the first generation of PWHIV in the country who acquired HIV from heterosexual contact. After they were diagnosed, delays in accessing treatment during that period resulted in immunosuppression. After starting ART, they have continuously been receiving HIV care through the Thai national programme for over a decade. The results revealed the presence of NCI of varying severity, which highlights the need for monitoring, intervention and social support. The findings from this study can help inform policymakers and may encourage more budget allocations to tailor health programmes for OAHIV. Our study limitations include enrolment bias, as only OAHIV who visited the clinics were included, in which case those with profound dementia or extreme QOL might not be included. Second, there were some participants with low literacy for whom MoCA might not be the optimal screening tool. However, we chose this instrument because of its widespread use in Thailand, which allowed us to make comparisons with other studies in Thai OAHIV. Third, we did not include data on all historic or current opportunistic infections and comorbidities, which could have been the cause of chronic inflammation or affected the central nervous system function. Fourth, we did not assess NCI in older adults without HIV of comparable age, sex, education level and geographic location for comparison. This limited our ability to make conclusions regarding the effects of such factors on the study results.

## 5 CONCLUSIONS

We documented NCI in a large majority of OAHIV with immune recovery and virologic suppression following delayed ART initiation. Further assessment of functioning to address problems in performing activities of daily living is required in those with low scores. More research to identify potentially modifiable factors affecting neurocognitive performance in ageing populations who started ART both later and sooner after HIV acquisition is warranted. Early detection of NCI among this population would allow healthcare providers to monitor, counsel or intervene appropriately in a more timely manner.

## COMPETING INTERESTS

The authors declare that they have no competing interests.

## AUTHORS’ CONTRIBUTIONS

LA, AT and KS designed the study. LA, WC and SS collected data and performed study assessments. PS and LA did the data analysis. LA and PS prepared the first draft of the manuscript. AT and KS reviewed and edited the main manuscript text. WC and SS composed the table. All authors reviewed the manuscript.

## FUNDING

The study was supported by the Center of Excellence in HIV/AIDS Chiang Mai University Research and the National Research University Project under Thailand's Office of the Higher Education Commission.

## Data Availability

The data that support the findings of this study are available from the corresponding author upon reasonable request.
